# Risk Factors for Hemorrhoids on Screening Colonoscopy

**DOI:** 10.1371/journal.pone.0139100

**Published:** 2015-09-25

**Authors:** Anne F. Peery, Robert S. Sandler, Joseph A. Galanko, Robert S. Bresalier, Jane C. Figueiredo, Dennis J. Ahnen, Elizabeth L. Barry, John A. Baron

**Affiliations:** 1 University of North Carolina School of Medicine, Chapel Hill, NC, United States of America; 2 The University of Texas MD Anderson Cancer Center, Houston, TX, United States of America; 3 Keck School of Medicine, University of Southern California, Los Angeles, CA, United States of America; 4 University of Colorado Anschutz Medical Campus, Denver, CO, United States of America; 5 Geisel School of Medicine at Dartmouth, Hanover, NH, United States of America; University Hospital Llandough, UNITED KINGDOM

## Abstract

**Background:**

Constipation, a low fiber diet, sedentary lifestyle and gravidity are commonly assumed to increase the risk of hemorrhoids. However, evidence regarding these factors is limited. We examined the association between commonly cited risk factors and the prevalence of hemorrhoids.

**Methods:**

We performed a cross sectional study of participants who underwent a colonoscopy in a colorectal adenoma prevention trial and who had a detailed assessment of bowel habits, diet and activity. The presence of hemorrhoids was extracted from the subjects’ colonoscopy reports. We used logistic regression to estimate odds ratios and 95% confidence intervals while adjusting for age and sex.

**Results:**

The study included 2,813 participants. Of these, 1,074 had hemorrhoids recorded. Constipation was associated with an increased prevalence of hemorrhoids (OR 1.43, 95% CI 1.11, 1.86). Of the fiber subtypes, high grain fiber intake was associated with a reduced risk (OR for quartile 4 versus quartile 1 = 0.78, 95% CI 0.62, 0.98). We found no association when comparing gravid and nulligravida women (OR 0.93, 95% CI 0.62–1.40). Sedentary behavior was associated with a reduced risk (OR 0.80, 95% CI 0.65–0.98), but not physical activity (OR 0.83, 95% CI 0.66–1.03). Neither being overweight nor obese was associated with the presence of hemorrhoids (OR 0.89, 95% CI 0.72–1.09 and OR 0.86, 95% CI 0.70–1.06).

**Conclusions:**

Constipation is associated with an increased risk of hemorrhoids. Gravidity and physical activity do not appear to be associated. High grain fiber intake and sedentary behavior are associated with a decreased risk of hemorrhoids.

## Introduction

Hemorrhoids are distal displacement and venous distention of the hemorrhoidal cushions,[1] a condition that affects 39 to 52% of adults.[2, 3] Hemorrhoids are commonly asymptomatic but can be complicated by painless bleeding, prolapse, soiling and pruritus ani.[1] In the United States, hemorrhoids are a common first-listed outpatient diagnosis with an estimated 2 million ambulatory clinic visits in 2004.[4] While there were nearly 2 million prescriptions for symptomatic hemorrhoids in 2004, most patients are thought to self-treat with over-the-counter therapy.[4] Surgery and endoscopic therapy for symptomatic hemorrhoids is common but the true burden of disease is difficult to capture.[4]

Risk factors for hemorrhoids remain poorly studied. Hemorrhoids are hypothesized to result from deterioration of anchoring connective tissue, prolapse of hemorrhoidal tissue, distention of the hemorrhoidal arteriovenous anastomoses or dilation of the veins of the internal hemorrhoidal venous plexus.[1] Factors commonly assumed to increase the risk of developing hemorrhoids include inadequate dietary fiber, constipation, diarrhea, chronic straining during defecation, pregnancy and a sedentary lifestyle, but these have actually not been well investigated.[4]

To explore factors associated with hemorrhoids, we analyzed data from colonoscopy reports that were collected as part of a chemoprevention study of colorectal adenomas. To learn more about potential risk factors for hemorrhoids, we considered multiple possible risk factors including bowel habits, diet, tobacco use, NSAID use, aspirin use, physical activity or lack thereof, body mass index, and obstetrical history. We hypothesized that a low fiber diet, constipation, straining with defecation, diarrhea, sedentary behavior, obesity, multigravida and live births would be associated with an increased risk of hemorrhoids on colonoscopy.

## Methods

### Study Design

This is a cross-sectional study using baseline data collected during a randomized clinical trial, the Vitamin D/Calcium Polyp Prevention study (ClinicalTrials.gov ID NCT00153816). This trial was a placebo-controlled trial of vitamin D and/or calcium supplementation for the prevention of colonic adenomas. Participants were recruited from eleven centers in the United States and Puerto Rico between July 2004 and July 2008. Eligible participants were between the ages of 45 and 75 with satisfactory preparation for colonoscopy, a complete exam to the cecum and at least one colorectal adenoma removed in the four months prior to study entry. The trial excluded individuals with a history of large bowel resection, a diagnosis of a familial colorectal cancer syndrome, inflammatory bowel disease, invasive colon cancer, or severe lung, heart, kidney or liver disease. The study was approved by the Institutional Review Board (IRB) of each study center including: Dartmouth College Committee for the Protection of Human Subjects, University of Southern California IRB, Kaiser Permanente IRB, Colorado Multiple IRB, Emory University IRB, Atlanta Veterans Affairs Medical Center (VAMC) IRB, University of Iowa IRB, University of Minnesota IRB, Minneapolis VAMC IRB, Portsmouth Regional Hospital IRB, Concord Hospital IRB, Maine Medical Center IRB, White River Junction VAMC IRB, University of North Carolina IRB, Cleveland Clinic IRB, University of Puerto Rico IRB, San Juan VAMC IRB, Palmetto Health IRB, University of Texas/MD Anderson Cancer Center IRB. Participants in the parent study provided written informed consent. Secondary data analysis of hemorrhoid and exposure data included no direct patient identifiers and was exempt from Institutional Review Board review.

### Study Population

All trial participants who completed the baseline visit were included. A research assistant trained in data abstraction and blinded to the exposure variables abstracted data from the qualifying colonoscopy reports. Cases were participants reported to have hemorrhoids; controls were subjects with no mention of hemorrhoids. Each participant had an intake visit for the parent study within 120 days after the colonoscopy. The intake visit collected information on demographics, diet, bowel habits, physical activity, smoking history, alcohol use, prescription and over the counter medication use. Dietary information was collected using the Block Brief 2000 Food Frequency Questionnaire, a semi-quantitative food frequency questionnaire with 60 food items.[5]

Bowel movement frequency was assessed with the question “In the past year, on average, how many bowel movements did you have [per day or per week]?” Bowel habits were further evaluated by inquiring what percent of the time a participant had to strain during a bowel movement, had a feeling of incomplete bowel evacuation, or had hard or lumpy stools.

Two definitions of constipation were used. The first definition was based on Rome I Criteria[6, 7] and was defined as having two or more of the following: 1) straining during a bowel movement 25% or more of the time, 2) a feeling of incomplete bowel evacuation 25% or more of the time, 3) having hard or lumpy stools 25% or more of the time, and/or 4) reporting fewer than three bowel movements per week. The second definition was frequency based and was defined as fewer than three bowel movements per week on average.

Physical activity was measured using the validated International Physical Activity Questionnaire,[8] and summarized as metabolic equivalents. Sedentary behavior was assessed with the question, “During the last 7 days, how much time did you usually spend sitting on a week day?” Height and weight were either measured (70% and 72.3%, respectively) or collected by self-report (29.6% and 24%, respectively) at this visit.

Regular use of aspirin or NSAIDS was defined as one or more days of use per week. Smoking was categorized as never, former and current. Alcohol use was assessed with number of alcoholic drinks per day over the last year. Pregnancy history was assessed with the questions “How many times have you been pregnant?” and “How many live births have you had?”

### Statistical Analysis

Means and standard deviations were reported for continuous variables, medians for skewed distributions of continuous variables, and proportions for categorical data. Characteristics were compared using a 2 sample t-test or Pearson’s chi-square test. Dietary data, alcohol use, physical activity and sedentary behavior were converted into quartiles for analyses. Multivariable analyses were performed using logistic regression to estimate odds ratios and 95% confidence intervals while adjusting for age, sex and constipation (when appropriate). Intake of total dietary fiber was adjusted for total caloric intake using regression residuals.[9] All tests of significance were two-tailed and p-values <0.05 were considered significant. The analysis was performed using SAS 9.2 (SAS, Cary, North Carolina).

## Results

Our analysis included 2,813 enrolled study participants. Of these, 1,074 (38%) had hemorrhoids noted in their colonoscopy report. Participants with hemorrhoids were more likely to be older, non-white, and less educated than those without. They reported fewer minutes sitting on a weekday and were more likely to meet criteria for constipation than controls (Table 1). There was no statistically significant difference among women in the mean number of total pregnancies (2.7 ± 1.8 versus 2.8 ± 1.8) or live births (2.4 ± 1.4 versus 2.4 ± 1.3).

**Table 1:** Population Characteristics.

	Hemorrhoids	No Hemorrhoids
	n = 1,074	n = 1,739
Age, years, mean ± SD***	58.7 ± 7.2	57.6 ± 6.7
Sex, n (%)		
Male	646 (60)	1,095 (63)
Female	428 (40)	644 (37)
Race, n (%)*		
White	843 (84)	1,445 (87)
Non-white	157 (16)	214 (13)
Education, years, mean ± SD***	11.2 ± 3.1	11.6 ± 2.8
BMI, kg/m^2^, mean ± SD	29.1 ± 5.5	29.2 ± 5.2
NSAID use (≥1 day/week), n (%)	247 (23)	436 (25)
Aspirin use (≥1 day/week), n (%)	441 (41)	687 (40)
Smoking status, n (%)		
Never	548 (51)	911 (52)
Former / Occasional	408 (38)	651 (37)
Current	117 (11)	177 (10)
Daily alcohol use, drinks/day, mean ± SD	0.77 ± 1.1	0.71 ± 1.0
Total energy intake, kcal/day, mean ± SD	1579 ± 587	1575 ± 587
Total dietary fiber, grams/day, mean ± SD	14.9 ± 7.1	15.1 ± 6.8
Supplemental fiber, n (%)	59 (5)	99 (6)
Physical activity per week, METS, mean ± SD	4125 ± 3985	4238 ± 3906
Sedentary behavior, minutes sitting on weekday, mean ± SD***	370 ± 198	397 ± 196
Constipation, Rome definition, n (%)**	127 (12)	145 (8)
Constipation, frequency definition, n (%)	21 (2)	24 (1)
Symptoms of constipation, n (%)		
Strain with bowel movement, ≥ 25%***	136 (13)	141 (8)
Incomplete bowel empty feeling, ≥ 25%*	135 (13)	176 (10)
Hard or lumpy stool, ≥ 25%*	174 (16)	230 (13)
Bowel movements per week, mean ± SD	9.0 ± 4.8	9.0 ± 4.9
Laxative use/last year, n (%)	83 (8)	139 (8)

Abbreviations: SD, standard deviation; BMI, body mass index; NSAID, nonsteroidal

anti-inflammatory drug; MET, metabolic equivalent task

Significance at the p<0.05 is indicated with a *

<0.01 with **

and <.001 with ***

A greater proportion of hemorrhoid cases met criteria for constipation defined by Rome I than controls (12% versus 8%, p = 0.002; OR 1.43, 95% CI 1.11, 1.86) (Tables 1 and 2). Similar proportions of cases and controls reported laxative use (8% versus 8%, p = 0.8; OR 0.93, 95% CI 0.70, 1.24)) (Tables 1).

**Table 2:** Assessment of Constipation and Related Symptoms as Risk Factors for Hemorrhoids.

	n	OR (95% CI)^1^
Constipation, Rome I definition		
No	2541	referent
Yes	272	1.43 (1.11–1.86)
Constipation, frequency definition		
≥3 Bowel movements per week	2768	referent
<3 Bowel movements per week	45	1.42 (0.78–2.58)
Strain during bowel movement		
Less than 25% of the time	2524	referent
25% or more of the time	277	1.58 (1.23–2.03)
Feeling of incomplete bowel movement		
Less than 25% of the time	2489	referent
25% or more of the time	311	1.27 (1.00–1.61)
Hard or lumpy stool		
Less than 25% of the time	2391	referent
25% or more of the time	404	1.28 (1.03–1.58)

^1^ Adjusted for sex and age

More cases than controls reported straining during bowel movements (13% versus 8%, p<0.0001; OR 1.58, 95% CI 1.23, 2.03), incomplete bowel empting during defecation (13% versus 10%, p = 0.04; OR 1.27, 95% CI 1.00, 1.61) and hard stools (16% versus 13%, p = 0.03; OR 1.28, 95% CI 1.03, 1.58) (Tables 1 and 2). However, frequent bowel movements were not associated with an increased risk. Compared to participants having 7 bowel movements per week, neither participants having 8–14 bowel movements per week (OR 0.97, 95% CI 0.80, 1.17) nor participants having 15 or more movements per week (OR 1.22, 95% CI 0.87, 1.70) had an increased odds of hemorrhoids.

There was no statistically significant difference between the cases and the controls in mean dietary fiber intake (14.9 grams versus 15.1 grams per day, p = 0.6) or in reported supplemental fiber intake (5% versus 6%, p = 0.8) (Table 1). Nonetheless, high total dietary fiber intake trended towards an association with a reduced risk of hemorrhoids (OR 0.80, 95% CI 0.64, 1.01) in a comparison of the highest quartile of fiber intake (mean 24.5 grams/day) to the lowest (mean 7.9 grams/day; p for trend over quartiles = 0.03) (Table 3). Of the fiber subtypes, high grain fiber intake was associated with a reduced risk (OR for quartile 4 vs. quartile 1 = 0.78, 95% CI 0.62, 0.98, p for trend over quartiles 0.15) (Table 3). We found no associations between intake of bean fiber or fruit/vegetable fiber and the presence of hemorrhoids (Table 3). Adjustment for constipation did not change these estimates significantly (data not shown).

**Table 3:** Assessment of Activity and Dietary Fiber as Risk Factors for Hemorrhoids.

	Quartile				
	1	2	3	4	p for trend
Physical activity					
OR (95% CI)^1^	referent	0.92 (0.74–1.15)	0.96 (0.78–1.19)	0.83 (0.66–1.03)	0.18
Sedentary behavior					
OR (95% CI)^1^	referent	0.89 (0.72–1.09)	0.82 (0.66–1.02)	0.80 (0.65–0.98)	0.02
Total dietary fiber, g/day					
OR (95% CI)^1^	referent	0.86 (0.69–1.08)	0.81 (0.65–1.02)	0.80 (0.64–1.01)	0.03
Bean fiber, g/day					
OR (95% CI)^1^	referent	1.00 (0.80–1.25)	0.88 (0.70–1.10)	1.08 (0.86–1.35)	0.94
Grain fiber, g/day					
OR (95% CI)^1^	referent	0.90 (0.72–1.13)	1.00 (0.80–1.25)	0.78 (0.62–0.98)	0.15
Fruit and vegetable fiber, g/day					
OR (95% CI)^1^	referent	0.90 (0.71–1.12)	0.88 (0.70–1.11)	0.87 (0.69–1.09)	0.18

^1^ Adjusted for sex and age

Individuals with the highest quartile of sedentary behavior (mean 656 minutes/day) had a lower risk of hemorrhoids than those in the lowest quartile (mean 176 minutes/day) (OR 0.80, 95% CI 0.65–0.98; p for trend = 0.02) (Table 3). In contrast, neither physical activity nor being overweight or obese was associated with increased risk (Tables 3 and 4). Alcohol, smoking, NSAID and aspirin use were not associated with hemorrhoids (Table 4). There was also no association between gravidity or number of live births and risk in women (Table 5).

**Table 4:** Assessment of NSAIDS, ASA, BMI and Smoking as Risk Factors for Hemorrhoids.

	n	OR (95% CI)
NSAID Use^1^		
Never / Non-Regular User	2,127	referent
Regular user	683	0.90 (0.75–1.07)
Aspirin Use^1^		
Never / Non-Regular User	1,684	referent
Regular user	1,128	1.02 (0.87–1.20)
BMI^1^		
Normal	610	referent
Overweight	1,132	0.89 (0.72–1.09)
Obese	1,063	0.86 (0.70–1.06)
Cigarette smoking^1^		
Never	1,459	referent
Ever	1,353	1.05 (0.90–1.22)

Abbreviations: BMI, body mass index; NSAID, nonsteroidal anti-inflammatory drug

^1^ Adjusted for sex and age

**Table 5:** Assessment of Gravidity and Number of Live Births as Risk Factors for Hemorrhoids.

		Gravidity			
	Nulligravida	1	2	3	≥4
n	107	122	282	277	282
OR (95% CI)^1^	referent	0.87 (0.51–1.48)	0.97 (0.61–1.52)	1.1 (0.73–1.80)	0.74 (0.47–1.17)
		Number of Live Births			
	Nulligravida	1	2	3	≥4
n	107	156	382	230	142
OR (95% CI)^1^	referent	1.05 (0.64–1.74)	0.97 (0.63–1.50)	0.85 (0.53–1.36)	0.86 (0.51–1.44)

^1^ Adjusted for age

## Discussion

Using colonoscopy data from a large clinical trial recording detailed information about bowel habits, diet, body mass index, and personal habits, we found that constipation was associated with increased risk of having hemorrhoids. More specifically, straining during bowel movements, a feeling of incomplete bowel movements, and having hard or lumpy stools at least 25% of the time were all associated with an increased risk. A diet high in grain fiber was associated with a reduced risk of hemorrhoids even after adjusting for constipation. Contrary to expectations, however, we found that frequent bowel movements and gravidity were not associated with hemorrhoids, while sedentary behavior was associated with a reduced risk. Tobacco use, alcohol use, non-aspirin NSAID use, aspirin use, physical activity, and body mass index were not associated with risk.

There have been few rigorous studies of constipation as a risk factor for hemorrhoids. Johanson and Sonnenberg[10] compared the epidemiology of hemorrhoids and constipation in a study that utilized several national surveys. They found that, in contrast with hemorrhoids, constipation was more common with increasing age, among blacks, and among those with low socioeconomic status and less education. In follow up to this report, Delco et al.[11] published a case-control study using administrative data and found that codes for constipation *were* associated with an increased risk of codes for hemorrhoid disease.

Johanson and Sonnenberg later published a case-control study of 325 veterans who underwent proctoscopy.[3] Neither constipation defined by objective criteria (stool frequency or consistency or straining to defecate) nor subjective criteria was associated with the presence of hemorrhoids. Riss et al.[12] published a cross-sectional study of 976 participants who had a colonoscopy and found that constipation was associated with an increased risk of hemorrhoids. Unfortunately, their group did not adjust for confounding variables

There have been few studies of straining with defecation and hemorrhoids. Johannsson and Sonnenberg[13]published a case control study of 100 patients who participated in a randomized study of hemorrhoidectomy who were compared to 100 population controls and 100 hospital controls Patients with hemorrhoids were more likely to report excessive straining and a feeling of incomplete emptying but there was no difference among the groups in bowel movement frequency. Controls did not have an anorectal exam, raising the possibility of asymptomatic hemorrhoids in the control group.

Several trials have found that dietary fiber is an effective treatment for symptomatic hemorrhoids.[14] We found that a diet high in grain fiber was associated with a reduced risk of hemorrhoids. We hypothesize that a high fiber diet reduces the risk of constipation, which is associated with hemorrhoids. Surprisingly, the association between high fiber intake and reduced risk of hemorrhoids held even after adjustment for constipation.

A subjective report of diarrhea was associated with an increased risk of hemorrhoids in a single case-control study.[3] The same study found no association between frequent or watery stools and hemorrhoids. We also found no association between more frequent bowel movements and hemorrhoids.

Hemorrhoids commonly complicate pregnancy. An estimated 85% of pregnant women in their second and third trimester have hemorrhoids. Whether a history of pregnancy or live births subsequently increases the risk of hemorrhoids following pregnancy is less well understood. The current obstetric literature predicts that hemorrhoids regress postpartum, but do not resolve completely.[15] Both the study by Riss et al[2] and our study found no association between gravidity and hemorrhoids, which suggests that hemorrhoids resolve postpartum.

Obesity was associated with hemorrhoids in two of the aforementioned studies [2, 3] but not in the present study. Despite the widespread belief that sitting for long periods is a risk factor for hemorrhoids, we found that sedentary behavior was associated with a reduced risk. Sedentary behavior was one component of the International Physical Activity Questionnaire but there was no indication that total physical activity per se was associated with hemorrhoids.

In contrast to prior work, our analysis was performed using a large, well-characterized population that had a complete colonoscopy for the purpose of colorectal screening or adenoma surveillance. We utilized a well-accepted definition of constipation,[16] and confounding variables were included in our models. We also included in our analysis a frequency-based definition of constipation and assessed the symptoms of constipation independently. While we found no statistically significant association between hemorrhoids and constipation defined by frequency, stool frequency is thought to be a poor measure of constipation.[17]

There are limitations to our study. This is a cross-sectional study of individuals with a recent history of colorectal adenomas and is therefore not representative of the general population. A detailed exam and reporting of the anorectum was not required in the clinical trial, although many endoscopists consider a detailed exam of the rectum and anal canal to be standard of care.[18] Furthermore, an examination with an anoscope is the gold standard for diagnosing hemorrhoids and excess air insufflation during retroflexion of the colonoscopy may have resulted in false negative results. Moreover, we did not have any information about the grade or type of hemorrhoids. It is reassuring that the prevalence of hemorrhoids in our population (38%) is similar to prevalence estimates in similar populations (39%).[2]

The present study examined a number of risk factors that have been classically associated with hemorrhoids, specifically: low-fiber diet, straining with defecation, constipation, diarrhea, gravidity, and sedentary behavior. In a large, well-characterized population, only three of these six risk factors were supported by the data. Our work supports the common wisdom that a low-fiber diet, straining with defecation and constipation are associated with an increased risk of hemorrhoids. However, contrary to expectation, diarrhea and gravidity were not associated with hemorrhoids, and sedentary behavior was associated with a reduced risk.
